# Na_V_1.7: Stress-induced changes in immunoreactivity within magnocellular neurosecretory neurons of the supraoptic nucleus

**DOI:** 10.1186/1744-8069-9-39

**Published:** 2013-08-08

**Authors:** Joel A Black, Janneke GJ Hoeijmakers, Catharina G Faber, Ingemar SJ Merkies, Stephen G Waxman

**Affiliations:** 1 Department of Neurology, Yale University School of Medicine, New Haven, CT 06510, USA; 2 Center for Neuroscience and Regeneration Research, Veterans Affairs Connecticut Healthcare System, West Haven, CT USA; 3 Department of Neurology, University Medical Center Maastricht, Maastricht, the Netherlands; 4 Department of Neurology, Spaarne Hospital, Hoofddorp, the Netherlands

**Keywords:** Hypothalamus, Na_v_1.7, Salt-loading, Supraoptic nucleus

## Abstract

**Background:**

Na_V_1.7 is preferentially expressed, at relatively high levels, in peripheral neurons, and is often referred to as a “peripheral” sodium channel, and Na_V_1.7-specific blockers are under study as potential pain therapeutics which might be expected to have minimal CNS side effects. However, occasional reports of patients with Na_V_1.7 gain-of-function mutations and apparent hypothalamic dysfunction have appeared. The two sodium channels previously studied within the rat hypothalamic supraoptic nucleus, Na_V_1.2 and Na_V_1.6, display up-regulated expression in response to osmotic stress.

**Results:**

Here we show that Na_V_1.7 is present within vasopressin-producing neurons and oxytocin-producing neurons within the rat hypothalamus, and demonstrate that the level of Na_v_1.7 immunoreactivity is increased in these cells in response to osmotic stress.

**Conclusions:**

Na_V_1.7 is present within neurosecretory neurons of rat supraoptic nucleus, where the level of immunoreactivity is dynamic, increasing in response to osmotic stress. Whether Na_V_1.7 levels are up-regulated within the human hypothalamus in response to environmental factors or stress, and whether Na_V_1.7 plays a functional role in human hypothalamus, is not yet known. Until these questions are resolved, the present findings suggest the need for careful assessment of hypothalamic function in patients with Na_V_1.7 mutations, especially when subjected to stress, and for monitoring of hypothalamic function as Na_V_1.7 blocking agents are studied.

## Background

Gain-of-function mutations of the Na_V_1.7 sodium channel, which is preferentially expressed at relatively high levels within peripheral (dorsal root ganglion and sympathetic ganglion) neurons [1-3] produce several syndromes associated with severe pain, including inherited erythromelalgia [4-8] and paroxysmal extreme pain disorder [9,10] as well as painful small-fiber neuropathy [11,12], while loss-of-function mutations of Na_V_1.7 cause channelopathy-associated insensitivity to pain [13-15]. In contrast with the severe pain associated with gain-of-function mutations of Na_V_1.7 and loss of pain sensitivity associated with loss-of-function mutations of Na_V_1.7, abnormalities of CNS function have in general not been reported in these disorders, consistent with preferential expression of Na_V_1.7 within peripheral neurons. Na_V_1.7-specific blockers are being studied as potential therapies for pain, with the rationale that they would be expected to have few, if any, CNS-related side-effects. Nevertheless, there have been reports of hypothermia, possibly due to an abnormality of central (hypothalamic) thermoregulation [16-18] in patients with Na_V_1.7 mutations and erythromelalgia. The syndrome of inappropriate release of antidiuretic hormone, SIADH, without any structural cause, recently developed in a patient carrying a gain-of-function mutation of Na_V_1.7, G856D, within a kindred with painful small-fiber neuropathy (Hoeijmakers et al, personal communication). Affected family members, all of whom carry the G856D mutation, display small-fiber neuropathy characterized by severe pain and vasomotor dyscontrol in their distal extremities, small hands and feet, and autonomic dysfunction. The G856D mutation enhances channel activation, impairs fast-inactivation, and markedly enhances the channel’s persistent current and response to slow ramp stimuli. The occurrence of SIADH in this patient suggested the possibility that the gain-of-function mutation in Na_V_1.7 might have contributed to hyperexcitability of vasopressin-releasing (magnocellular neurosecretory) neurons in the supraoptic nucleus within the hypothalamus.

Vasopressin release by supraoptic magnocellular neurons can be triggered by osmotic stress and depends on bursting activity in these cells [19]. It is known that tetrodotoxin-sensitive sodium channels contribute to this bursting [20-22]. While high levels of expression of Na_V_1.7 have been reported in hypothalamic nuclei including the supraoptic nucleus in rodents [13,23], only weak levels of Na_V_1.7 expression were detected within the primate supraoptic nucleus [13]. In the present study, we have built upon earlier studies in rodents which showed that the deployment of sodium channels in the hypothalamus is dynamic, with levels of expression of the two sodium channel subtypes that were previously studied, Na_V_1.2 and Na_V_1.6, and of sodium channel beta-1 and beta-2 subunits and sodium currents, displaying up-regulation within supraoptic magnocellular neurons exposed to osmotic stress via salt-loading [24] and as a result of the hyperosmolar state associated with experimental diabetes [25]. Reasoning that Na_v_1.7 expression within supraoptic magnocellular neurons might be subject to similar plasticity, we exposed rats to salt-loading and assessed the level of Na_V_1.7 immunoreactivity within these neurons. We demonstrate here that Na_V_1.7 is present within vasopressin- and oxytocin-producing neurons of the supraoptic nucleus, and show that the level of Na_V_1.7 protein in these cells in not static but, on the contrary, is increased in response to salt-loading.

## Results

Previous work from our laboratory has demonstrated the expression of the tetrodotoxin-sensitive (TTX-S) sodium channels, Na_V_1.2 and Na_V_1.6, but not Na_V_1.1 and Na_V_1.3, and of TTX-S sodium currents in magnocellular neurosecretory cells (MSC) of the hypothalamic supraoptic nucleus [24]. This early study also showed that the expression of Na_V_1.2 and Na_V_1.6 channels are upregulated and amplitude of the sodium current increased following salt-loading challenge [24]. To determine whether Na_V_1.7 is expressed and upregulated in magnocellular neurosecretory cells of the supraoptic nucleus, we assessed the supraoptic nucleus of control and salt-loaded (2% NaCl in drinking water) rats using immunocytochemistry. Measurement of plasma osmotic pressure confirmed the presence of hyperosmolarity in the salt-loaded rats: control, 323.3 ± 4.8 mOsm; salt-loaded, 353.2 ± 3.3 mOsm (p < 0.05).

**Figure 1:**
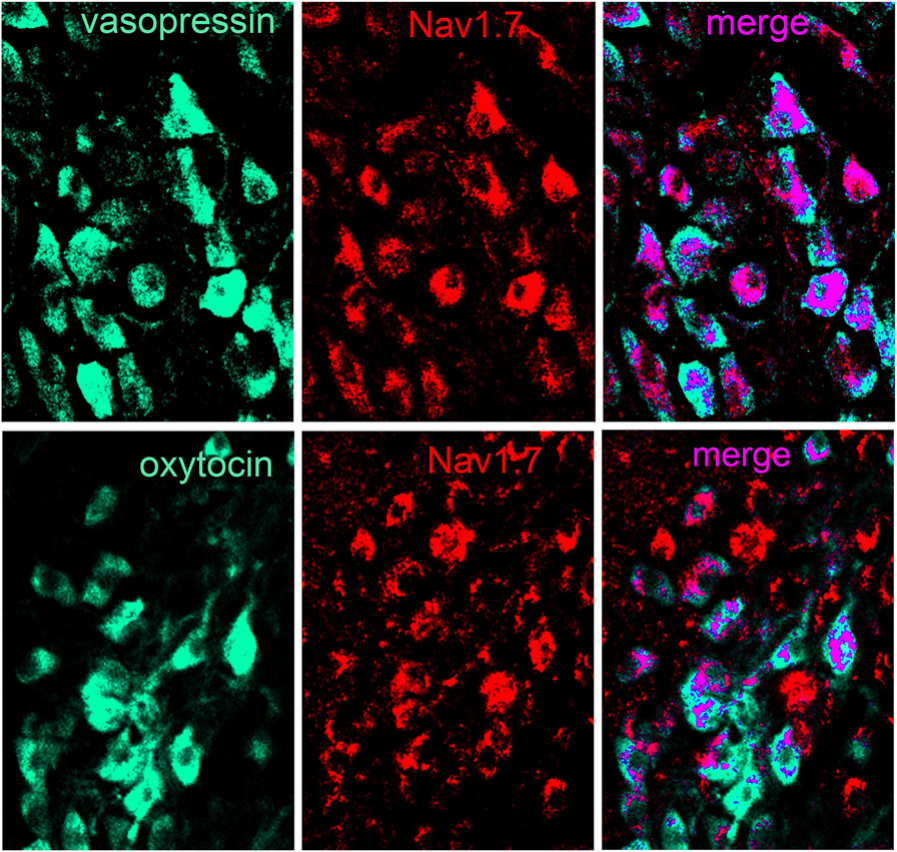
**Na**_**v**_**1.7 expression in vasopressin- and oxytocin-producing magnocellular neurosecretory cells in supraoptic nucleus.** Magnocellular neurosecretory neurons (MSN) of the supraoptic nucleus (SON) exhibit robust vasopressin and oxytocin immunolabeling (green). MSN of the SON display Na_v_1.7 immunoreactivity (red). Double-immunocytochemical studies with antibodies to vasopressin or oxytocin and Na_v_1.7 demonstrate that both peptide-producing cell-types exhibit co-localization (magenta) with Na_v_1.7. Merged image of vasopressin or oxytocin with Na_v_1.7 is presented as magenta to enhance visualization of co-localization.

Magnocellular neurosecretory cells in the supraoptic nucleus of control rats exhibited distinct Na_V_1.7 immunolabeling (Figure 1). Some magnocellular neurosecretory cells displayed moderate levels of Na_V_1.7 immunosignal, while other magnocellular neurosecretory cells exhibited a low level or no Na_V_1.7 immunofluorescence. Two types of magnocellular neurosecretory cells exist within the supraoptic nucleus, oxytocin-producing and vasopressin-producing, with little co-expression of these hormones in individual magnocellular neurosecretory cells. Both oxytocin- and vasopressin-producing magnocellular neurosecretory cells displayed Na_V_1.7 immunolabeling (Figure 1). Approximately 72% (33 of 46) of oxytocin-producing and 53% (59 of 112) of vasopressin-producing MSC expressed Na_V_1.7 labeling above background levels.

**Figure 2:**
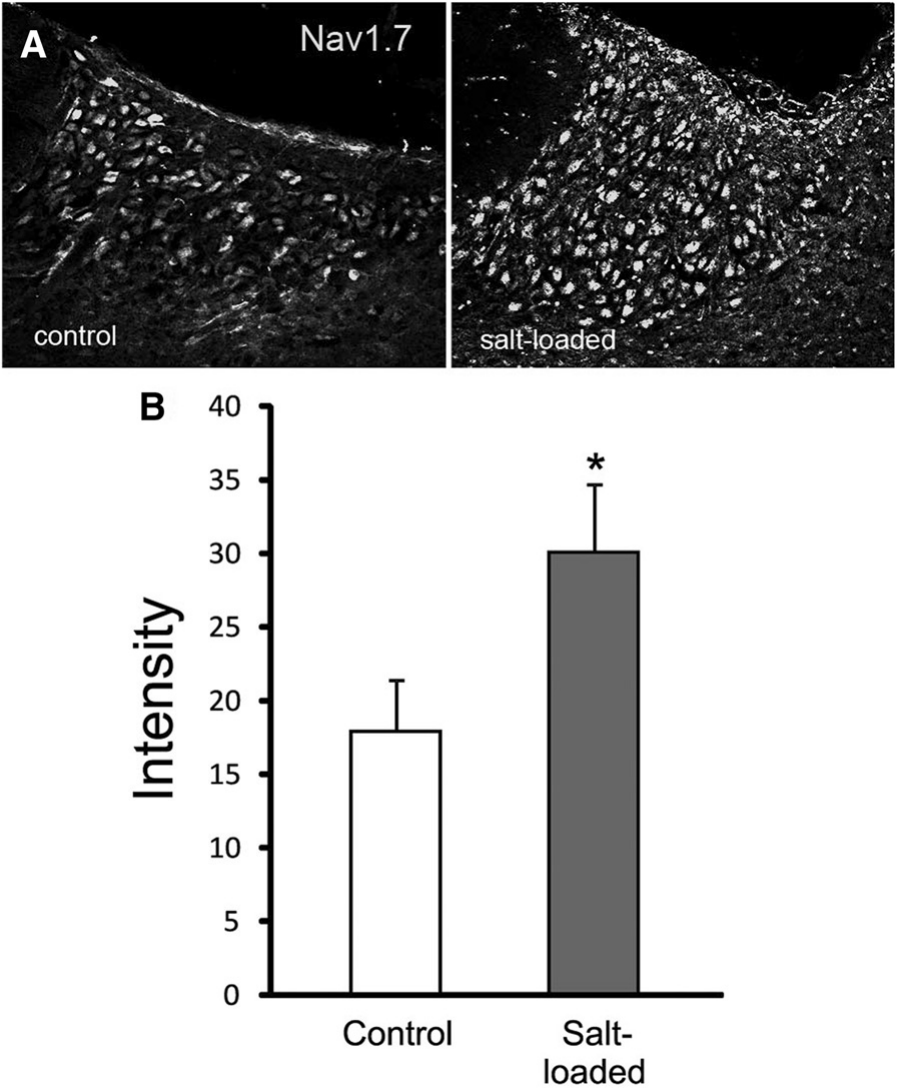
**Na**_**v**_**1.7 is upregulated in magnocellular neurosecretory neurons of supraoptic nucleus following salt loading. (A)** Magnocellular neurosecretory neurons (MSN) within supraoptic nucleus (SON) of control rats exhibit Na_v_1.7 immunolabeling. Following salt-loading, MSN display more prominent Na_v_1.7 immunoreactivity compared to control rats. **(B)** Quantification of Na_v_1.7 immunosignal within the SON demonstrates an approximately two-fold increase in Na_v_1.7 immunofluorescence in SON of salt-loaded rats compared to control rats.

Salt-loading induced a substantial increase in the level of Na_V_1.7 immunoreactivity in magnocellular neurosecretory cells of the supraoptic nucleus compared to magnocellular neurosecretory cells in control rats (Figure 2A). In addition to the detection of greater numbers of magnocellular neurosecretory cells that displayed Na_V_1.7 immunolabeling, the intensity of Na_V_1.7 signal in some magnocellular neurosecretory neurons was markedly greater than that observed in magnocellular neurosecretory cells from control rats. Quantification of the mean intensity of Na_V_1.7 signal within the circumscribed supraoptic nucleus demonstrated a significant up regulation of Na_V_1.7 in response to salt-loading challenge (Figure 2B). These observations demonstrate that, in addition to up regulation of the TTX-S sodium channels Na_V_1.2 and Na_V_1.6 within magnocellular neurosecretory cells of the supraoptic nucleus with salt-loading, the level of Na_V_1.7 protein in these cells is significantly increased in osmotically-challenged rats.

## Discussion

In this study we have demonstrated that Na_V_1.7 is present within neurons within the hypothalamic supraoptic nucleus, specifically within vasopressin- and oxytocin-producing magnocellular neurosecretory neurons. We also show that the level of Na_V_1.7 protein in these cells is not fixed but, on the contrary, is dynamic, increasing as a result of salt-loading.

A role Na_V_1.7 in electrogenesis in DRG neurons is well-established, and it is clear that Na_V_1.7 functions as a threshold channel in these neurons, amplifying small depolarizing inputs to bring the cell to threshold for action potential generation [26,27] and possibly facilitating invasion into, and/or transmitter release from, preterminal axons within the spinal cord dorsal horn [1,28]. In contrast, a functional role of Na_V_1.7 within supraoptic neurons is less well understood. Action potential bursts, triggered by osmotic changes, lead to release of vasopressin by supraoptic magnocellular neurons [19] and it is known that tetrodotoxin-sensitive sodium channels contribute to this bursting [20-22].

Supraoptic magnocellular neurons are known to be highly dynamic. It is known that, in response to changes in osmolality, the expression of peptides within these cells changes, and they change in size [29]. In parallel, it has been shown that in response to increased osmolarity there are changes in deployment of sodium channels, with up-regulated expression of the Na_V_1.2 and Na_V_1.6 alpha subunits, and of the sodium channel beta-1 and beta-2 subunits [24,25]. The present results show that the level of Na_V_1.7 protein, like that of Na_V_1.2 and Na_V_1.6 [24,25], is dynamic, and is up-regulated within supraoptic magnocellular neurons exposed to osmotic stress via salt-loading. A previous study [24] demonstrated an increase in the amplitude of the transient Na^+^ current, and an even greater increase in the amplitude and density of the Na^+^ currents evoked by slow ramp stimuli in supraoptic neurons following salt-loading. While definitive identification of the current as Na_V_1.7 current would require specific blockade or knockout, both of these types of current have been observed to be produced by Na_V_1.7 [26]. Because Na_V_1.7 is present within vasopressin neurons, it seems likely that this sodium channel isoform plays some role in vasopressin release in response to the osmotic stress imposed by salt-loading.

Although only low levels of Na_V_1.7 have been reported in the hypothalamus in primates [13], it is possible that the density of Na_V_1.7 channels within magnocellular neurons of the human supraoptic nucleus, like that in rodents, is subject to up-regulation in response to some forms of stress. Na_V_1.7 blockers are currently under development as potential pharmacotherapeutics for pain [30-34]. Hypothalamic dysfunction has not been observed thus far in families with channelopathy-associated insensitivity to pain due to null mutations in the gene encoding Na_V_1.7. However, functional Na_V_1.7 channels are absent beginning in early embryogenesis in affected individuals in these families, and the possibility that there might be compensatory changes in hypothalamic neurons which maintain relatively normal function in these cells cannot be excluded. Whether levels of Na_V_1.7 are increased in response to environmental factors or stress within the human hypothalamus, and whether Na_V_1.7 plays a functional role in hypothalamic neurons in humans, is not known. Until these questions are resolved, the present findings suggest the need for assessment of hypothalamic function in patients carrying Na_V_1.7 mutations especially when subjected to stress, and for monitoring of hypothalamic function as Na_V_1.7 blocking agents are studied.

## Conclusions

In summary, our results demonstrate that sodium channel Na_V_1.7 is expressed in vasopressin-producing and oxytocin-producing magnocellular neurosecretory neurons of the rat hypothalamic supraoptic nucleus. The level of Na_V_1.7 immunoreactivity in the supraoptic nucleus is significantly increased following salt-loading, suggesting a contribution of Na_V_1.7 in the response of magnocellular neurosecretory neurons to osmotic stress. While it is not yet known whether levels of expression of Na_V_1.7 are increased in response to environmental factors or stress within the human hypothalamus, or whether Na_V_1.7 plays a functional role in hypothalamic neurons in humans, the present findings suggest the need for assessment of hypothalamic function in patients carrying Na_V_1.7 mutations, especially when subjected to stress, and for monitoring of hypothalamic function as Na_V_1.7 blocking agents are studied.

## Methods

### Salt loading

Adult male Sprague-Dawley rats (200-220 g), housed under a 12 h-12 h dark-light cycle, were salt-loaded with 2% NaCl (ad libitum) in their drinking water and unlimited access to food. Rats were sacrificed for immunocytochemical investigation 7 days following salt loading. All experiments were approved by the VA Connecticut Healthcare System Institutional Animal Care and Use Committee. To confirm the extent of salt loading, plasma osmotic pressure of the rats was measured (vapor pressure osmometer model 5500, Wescor, USA). Body weights were significantly (p < .05) lower in salt-loaded (186.6 ± 4.7 g) compared to control (248.6 ± 1.9) rats. Six control and 6 salt-loaded rats were used for the immunocytochemistry studies.

### Immunocytochemistry

Rats were perfused with 4% paraformaldehyde in 0.14 M Sorensen’s phosphate buffer, pH 7.4, and the brain removed and postfixed for 25-30 minutes. After cryoprotection in 30% sucrose in 0.01 M PBS for 24 h, the brains were blocked, frozen and coronal cryosections (16 μm) containing SON and optic chiasm were cut. Sections were incubated in blocking solution (PBS containing 3% normal donkey serum, 3% fish skin gelatin, 0.1% Triton X-100 and 0.02% sodium azide) for 15 min at room temperature, primary antibody(ies) [rabbit anti- Na_V_1.7, 1:200, Y083 (Black et al. 2012); guinea pig anti-vasopressin, 1:100, Peninsula Lab, San Carlos, CA); mouse anti-oxytocin, 1:500, Abcam, Cambridge, MA)] in blocking solution 2-4 days at 4°C, rinsed in PBS, incubated 1-2 days at 4°C with secondary antibody(ies) [donkey anti-rabbit Alexa Fluor-488-congugated F(ab’)_2_ fragment, donkey anti-rabbit Alexa Fluor-Cy3, donkey anti-guinea pig Alexa Fluor-488; donkey anti-mouse Alexa Fluor-488; all secondary antibodies from Jackson Immuno Research, West Grove, PA], rinsed with PBS and mounted on glass slides with Aqua-polymount (Polyscience, Warrington, PA). Control experiments in which the primary antibody was omitted exhibited only background levels of labeling.

### Quantification

Tissue sections were examined with a Nikon C1 confocal microscope (Nikon USA, Melville, NY), using a 20× objective and operating in single mode for detection of Na_V_1.7 alone or in frame lamba (sequential) mode for detection of Na_V_1.7 and vasopressin or oxytocin to prevent possible bleed-through between 488 and Cy3 channels.

For detection of Na_V_1.7 in the supraoptic nucleus (SON), images were acquired from 6 control and 6 salt-loaded rats, utilizing the same confocal settings for acquisition of Na_V_1.7 immunofluorescent signals. Images were opened in Nikon Elements and the mean signal intensity of the circumscribed SON was calculated by the software.

For co-localization of Na_V_1.7 and vasopressin or oxytocin in SON neurons, signals for Na_V_1.7 and vasopressin were thresholded at intensities 20% above background levels, and the percentage of vasopressin neurons expressing Na_V_1.7 was calculated. Data are presented as mean ± SEM and statistical analysis was performed with Excel Student’s t-test, with p < 0.05 considered significant.

## Abbreviations

DRG: Dorsal root ganglion; MSN: Magnocellular neurosecretory neurons; SIADH: Syndrome of inappropriate release of anti-diuretic hormone; SON: Supraoptic nucleus.

## Competing interests

The authors declare no competing interests.

## Authors’ contributions

JAB designed immunocytochemical experiments, acquired, analyzed and interpreted data, and participated in writing manuscript. JGJH participated in conception and design of experiments and to editing the manuscript. CGF participated in conception and design of experiments and to editing the manuscript. ISJM participated in conception and design of experiments and to editing the manuscript. SGW participated in design and interpretation of experiments and in writing the manuscript. All authors read and approved the final manuscript.
